# Comparison of Rifamycins for Efficacy Against *Mycobacterium avium* Complex and Resistance Emergence in the Hollow Fiber Model System

**DOI:** 10.3389/fphar.2021.645264

**Published:** 2021-04-15

**Authors:** Gunavanthi D. Boorgula, Laxmi U. M. R. Jakkula, Tawanda Gumbo, Bockgie Jung, Shashikant Srivastava

**Affiliations:** ^1^ Department of Pulmonary Immunology, University of Texas Health Science Center at Tyler, Tyler, TX, United States; ^2^ Quantitative Preclinical and Clinical Sciences Department, Praedicare Inc., Dallas, TX, United States; ^3^ Department of Immunology, UT Southwestern Medical Center, Dallas, TX, United States

**Keywords:** rifampin, rifapentine, rifabutin, pharmacokinetics/pharmacodynamics, resistance

## Abstract

Rifamycins are integral part of the combination regimen for treatment of pulmonary *Mycobacterium avium-complex* [MAC] infection, but different practitioners prefer different rifamycins. The objective of the study was to compare microbial kill and resistance emergence of rifamycins using principles of pharmacokinetics/pharmacodynamics. First, we identified rifamycin MICs in 20 MAC isolates from patients followed by concentration-response studies in test-tubes. Next, we examined efficacy and resistance suppression of three doses of each rifamycin in the hollow fiber system model of pulmonary MAC [HFS-MAC], mimicking human like concentration-time profile of the drugs. HFS-MAC units were repetitively sampled for total and drug-resistant MAC burden and for drug concentration measurements. Inhibitory sigmoid E _max_ model, linear regression, and analysis of variance was used for data analysis. For rifabutin 90% of isolates had MIC ≤ 0.125 mg/L while for both rifampin and rifapentine this was ≤2.0 mg/L. There was no statistically significant difference (*p* > 0.05) in maximal kill and effective concentration mediating 50% of the bacterial kill among three rifamycins in the static concentration experiment. In the HFS-MAC, the bactericidal kill (day 0–4) for rifampin was 0.89 (95% Confidence Interval (CI): 0.43–1.35), for rifapentine was 1.05 (95% CI: 0.08–1.23), and for rifabutin was 0.92 (95% CI: 0.61–1.24) log_10_ CFU/ml, respectively. Rifamycins monotherapy failed after just 4-days of treatment and entire MAC population was drug resistant on day 26 of the study. There was no dose dependent difference in MAC kill or resistance suppression among the three rifamycins tested in the HFS-MAC. Therefore, replacing one rifamycin, due to emergence of drug-resistance, with other may not be beneficial in clinical setting.

## Introduction

Nontuberculous mycobacteria (NTM), especially species of the *Mycobacterium avium* complex (MAC), are among the most difficult to treat pulmonary infection (Cowman et al., 2019). A recent retrospective study showed that between 2008 to 2015, the annual NTM lung disease incidence increased from 3.13 to 4.73 per 100,000 person, and the annual prevalence changed from 6.78 to 11.7 (Winthrop et al., 2020). During the same period, the prevalence among the women increased from 9.63 to 16.7 per 100,000, while among those 65 years or older it increased from 30.27 to 47.48 per 100,000 (Winthrop et al., 2020). The current American Thoracic Society [ATS] guidelines recommend a macrolide-ethambutol-rifamycin combination therapy for treatment of pulmonary MAC (Griffith, 2007). However, as per a recent meta-analyses, despite 18–23 months long therapy duration, the sustained sputum culture conversion rates at the end of therapy, a marker of cure, were only 54% (Pasipanodya et al., 2017). Just as clinicians use different macrolides (azithromycin or clarithromycin], they also use different rifamycins [rifampin or rifabutin). It is unclear which rifamycin is better; moreover, how rifapentine would fare in treatment of pulmonary MAC is currently unclear. We compared the three rifamycins head-to-head using pharmacokinetics/pharmacodynamic (PK/PD) study design.

We have developed a pre-clinical hollow fiber system model of pulmonary MAC (HFS-MAC), that can mimic the human like PK of the drugs (Deshpande et al., 2010a; Deshpande et al., 2010b; Deshpande and Gumbo, 2011; Deshpande et al., 2016b; Deshpande et al., 2017a; Deshpande et al., 2017b; Deshpande et al., 2017c; Deshpande et al., 2017d; Srivastava et al., 2017a; Srivastava et al., 2017b). We have used the HFS-MAC for PK/PD studies with azithromycin, ethambutol, moxifloxacin, thioridazine, linezolid, tedizolid, ceftazidime-avibactam, minocycline, and tigecycline (Deshpande et al., 2010a; Deshpande et al., 2010b; Deshpande et al., 2016a; Deshpande et al., 2016b; Deshpande et al., 2017a; Deshpande et al., 2017b; Deshpande et al., 2017d). In the present study, we utilized the same model system for rifamycins PK/PD that allowed us to compare how this class of drugs perform relative to other drugs used to treat pulmonary MAC infection.

## Materials and Methods

### Bacteria, Media, and Other Supplies

The study protocol was approved by the institutional infectious organism research review committee. All experiments were performed in the BSL2 laboratory. Prior to each experiment the stock culture of MAC (American Type Culture Collection, ATCC#700898 and 20 clinical isolates provided by Dr Barbara Brown-Elliot, Department of Microbiology, University of Texas Health Science Center at Tyler, TX) were grown to log phase growth at 37°C in Middlebrook 7H9 broth supplemented with 10% oleic acid-albumin-dextrose-catalase (OADC) [herein, termed “broth”] under shaking condition. Hollow fiber cartridges were purchased from FiberCell (Frederick, MD). Drugs were purchased from Sigma Aldrich. Drugs were first dissolved in 100% dimethyl sulfoxide [DMSO] followed by dilution in 25% DMSO to achieve the desired concentration to use in the experiments. The final concentration of DMSO was kept at <1%, having no effect on MAC growth.

### Determination of Minimum Inhibitory Concentration

MIC of rifampin, rifapentine, and rifabutin was determined using the broth micro-dilution method (CLSI, 2018). Briefly, bacteria (standard laboratory strain, ATCC#700898, as well as 20 clinical isolates) were grown to log-phase growth in broth to an optical density of 0.07–0.08 at a wavelength of 600 nm, corresponding to McFarland standard 0.5 (McFarland, 1907). The cultures were then diluted 100-fold in broth to achieve a bacterial density of ∼10^5^ CFU/ml. Next, 180 μL of the cultures were added to each well of 96-well plates, pre-filled with 20 μL of the drugs at 10x concentration (10-fold dilution). The final drug concentrations were 0 (non-treated), 0.03125, 0.0625, 0.125, 0.25, 0.5, 1.0, and 2 mg/L. The plates were sealed in plastic bags and cultures were incubated at 37°C under for 7 days. On day 7, the plates were read using an inverted mirror and MIC was defined as the lowest drug concentration that completely inhibited visible microbial growth in the wells. The experiment was performed twice with two replicates for each drug concentration.

### Rifamycins Concentration-Response Study in Test-Tubes

The experiment was performed only with the standard laboratory strain of MAC. The method for inoculum preparation was same as described above. The concentrations of rifampin, rifapentine, and rifabutin were 0 (non-treated), 0.03125, 0.0625, 0.125, 0.25, 0.5, 1.0, and 4 mg/L. The experiment was performed in triplicate with a total volume of 5 ml in 15 ml screw caped tubes. MAC cultures with each drug concentration were co-incubated for 7 days, followed by washing twice in normal saline to remove carry over drug, then 10-fold serial dilution and finally culture on Middlebrook 7H10 agar supplemented with 10% OADC (herein termed “agar”). The cultures were incubated at 37°C for 10–14 days before colony forming units (CFU) were recorded.

### Comparison of Rifamycins Using the Hollow Fiber Model System

The detailed description of the HFS-MAC has been published elsewhere (Deshpande et al., 2010a; Deshpande et al., 2010b; Srivastava and Gumbo, 2011; Srivastava et al., 2017a). To summarize, the central compartment of the HFS-MAC receives the drugs administered via computer-controlled syringe pump. The drug infusion rate determines the time (T_max_) to reach the peak concentration (C_max_) with given human equivalent dose. The continuous infusion of fresh media into the central compartment, at predetermined inflow rate, control the half-life (t_1/2_) of the drugs in the HFS-MAC. Next, a set of duet pumps circulate the drug containing media from the central compartment to the peripheral compartment, that house semipermeable hollow fiber membranes. The pore size can be selected in such a way it allows the nutrients and drugs to cross the membrane but keep the bacteria in the peripheral compartment. Finally, a second set of peristaltic masterflex pump removes the waste media from the central compartment. Thus, the HFS-MAC serves as a continuous dilution system where the bacteria always remain in contact with fluctuating drug concentration, Based on the previous publications, we used the free (*f*) drug concentration and resultant drug exposure of rifampin (Gumbo et al., 2007), rifapentine (Egelund et al., 2014), and rifabutin (Blaschke and Skinner, 1996; No_Author_Listed (2014). Mycobutin [Rifabutin] capsule, USP [Online]. Available: https://www.accessdata.fda.gov/drugsatfda_docs/label/2014/050689Orig1s018lbl.pdf [Accessed 10/20/2020 2020].) to treat the HFS-MAC units. The elsewhere reported protein binding of rifampin, rifapentine, and rifabutin were used to determine the clinically relevant drug exposures to test in the HFS-MAC (Gumbo et al., 2007; Dooley et al., 2012; Naiker et al., 2014). The HFS-MAC units were housed in incubators at 37°C under 5% CO_2_ and pre-conditioned with broth (where OADC was replaced with 10% dextrose) for 72 h. We used three different drug exposures, standard as well as high dose, rifamycins as shown in Table 1. (Gumbo et al., 2007; Dooley et al., 2012; Naiker et al., 2014). In the HFS-MAC, we mimicked 3 h half-life for rifampin and 15 h for rifapentine and rifabutin. The experiment was performed with one HFS-MAC system per drug exposure and three non-treated controls, thus a total of 12 HFS-MAC units.

**TABLE 1:** Rifampin, rifapentine, and rifabutin drug exposure achieved in the HFS-MAC.

Regimen	Drug	*f*C_max_ (mg/L)	*f*AUC_0-24_ (mg*hr/L)	*f*C_max_/MIC	*f*AUC_0-24_/MIC
RIF-1	Rifampin	0.76	5.79	26.54	192.97
RIF-2	Rifampin	2.03	19	67.63	633.33
RIF-3	Rifampin	4.12	39.84	137.2	1328
RFP-1	Rifapentine	0.15	1.38	5.1	46.1
RFP-2	Rifapentine	0.17	2.22	5.62	73.87
RFP-3	Rifapentine	1.02	13.05	33.93	435
RFB-1	Rifabutin	1.05	12.52	35	417.33
RFB-2	Rifabutin	1.98	26.91	66.07	897
RFB-3	Rifabutin	3.48	48.19	115.87	1606.33
CTL-1	Non-treated	–	–	–	–
CTL-2	Non-treated	–	–	–	–
CTL-3	Non-treated	–	–	–	–

Twenty mL of the log-phase growth cultures of MAC (ATCC#700898) were inoculated into the peripheral compartment of each HFS-MAC unit. The drugs were infused in the central compartment, over 1 h, using programable syringe pumps. The peripheral compartment of each HFS-MAC unit was sampled on days 0, 4, 7, 14, 21 and 26. The samples were washed twice with normal saline to remove carry over drug followed by 10-fold serial dilution, as described in detail previously (Deshpande et al., 2010a; Deshpande et al., 2010b; Srivastava et al., 2017a), to estimate the total bacterial burden on Middlebrook 7H10 agar. For enumeration of the drug resistant subpopulation, the same samples were cultured on agar containing 12x the MIC of each drug. The intent was not to capture the actual change in the MIC, hence single drug concentration was used. For the validation of the concentration-time profile of the drugs, the central compartment of each HFS-MAC unit was sampled on day 26. Thus, these represent steady state drug concentrations. The sampling time-points were as following: pre-dose, then at 1, 2, 3, 6 12, 18 and 23.5 h post dosing. We used previously described LC-MS/MS based concentration measurement assays, without modification (Gumbo et al., 2007; Deshpande et al., 2017c; Chapagain et al., 2020).

### Data Analysis

The measured drug concentrations were modeled using Phoenix WinNonlin 8.1 (Certara USA, Inc., MO, United States) and used to calculate the drug exposure in terms of ratio of peak concentration to MIC (C_max_/MIC) and 24 h area under the concentration-time curve to MIC (AUC_0–24_/MIC). GraphPad Prism (v 8.0) was used for graphing as well as to determine the relationship between the drug concentration and bacterial burden using the three-parameter (E_con_, E_max_, EC) inhibitory sigmoid E_max_ model (i.e., H fixed at 1), to perform the linear regression analysis to calculate the kill rate with each drug and dosing regimen, and to perform analysis of variance (ANOVA) to compare the different drug regimens.

## Results

**FIGURE 1:**
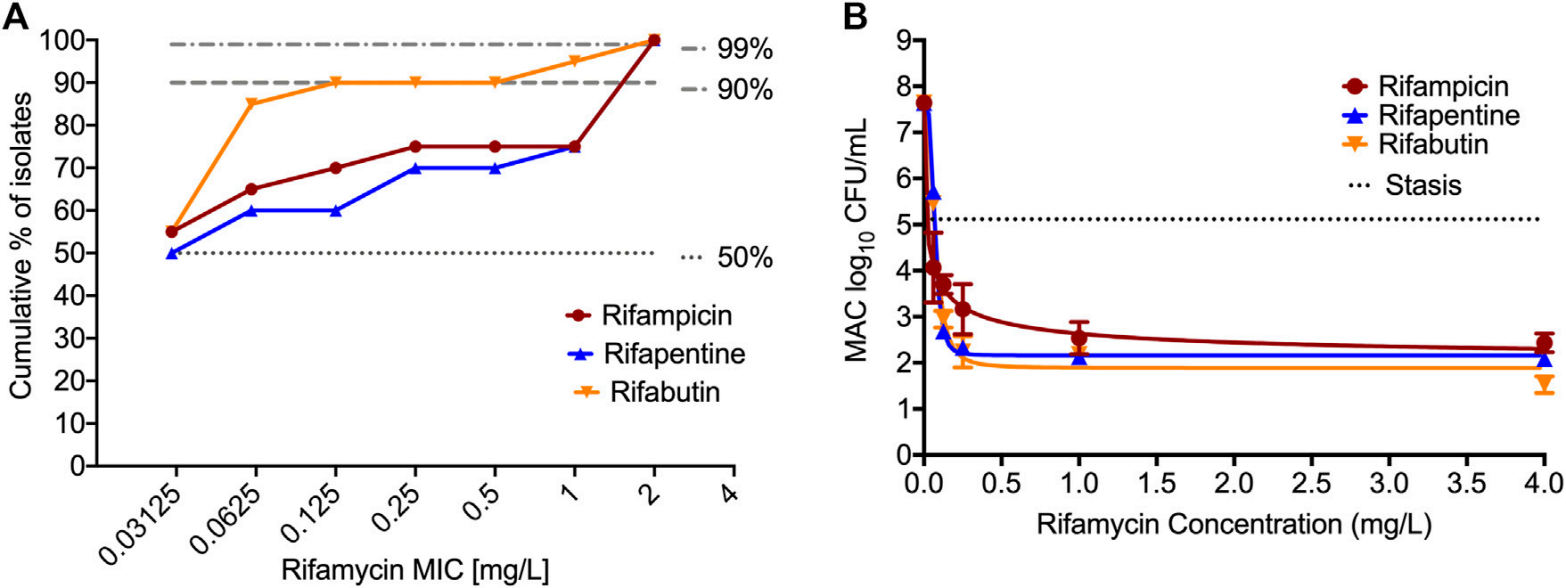
MIC distribution and concentration-response of rifamycins against MIC. **(A)**. MIC distribution of 20 MAC clinical isolates against the three rifamycins. **(B)**. Bacteria was exposed to different static concentrations of the drugs for seven days. The solid line is for model fit, and symbols represent the data point. The kill below stasis [day 0 or inoculum] with rifampin was 2.68 (95% Confidence Interval (CI): 1.13 to 4.24) log_10_ CFU/mL, for rifapentine was 3.03 (95% CI: 2.89 to 3.17) log_10_ CFU/mL, and for rifabutin was 3.59 (95% CI: 2.22 to 4.96) log_10_ CFU/mL, respectively.

Table 2 and Figure 1A show the rifamycin’s MIC distribution in the 20 clinical strains. The MIC_50_ for all isolates were at the lowest concentrations of drug tested i.e., 0.032 mg/L. The MIC_90_ of rifabutin was 5-tube dilutions and 16-fold times lower than rifampin and rifapentine. The MIC of the MAC laboratory strain used for all subsequent PK/PD studies was 0.032 mg/L for either rifampin, or rifapentine, or rifabutin, in two separate experiments with two replicate each. This isolate was then examined in the static concentration-response studies, with results shown in Figure 1B. The E_max_ (maximal kill) and EC_50_ (concentration mediating 50% of the E_max_) of rifampin were 5.674 (95% Confidence Interval (CI): 4.708–6.641) log_10_ CFU/mL and 0.023 (95% CI: 0.001 to 0.048) mg/L (*r* ^2^ = 0.972); for rifapentine were 5.480 (95% CI: 5.265 to 5.696) log_10_ CFU/mL and 0.070 (95% CI: 0.067 to 0.073) mg/L (*r* ^2^ = 0.998); and for rifabutin were 5.76 (95% CI: 5.189 to 6.329) log_10_ CFU/mL and 0.072 (95% CI: 061 to 0.082) mg/L (*r* ^2^ = 0.998), respectively. Therefore, the efficacy (E_max_) and potency (EC_50_) of the three rifamycins were virtually identical.

**TABLE 2:** MIC of *M. avium* clinical strains against rifamycins.

Strain ID	Rifampin	Rifapentine	Rifabutin
6824	0.125	0.06	0.06
65380	0.03	0.03	0.03
63064	0.03	0.03	0.03
63045	0.03	0.03	0.03
65195	0.06	0.25	0.06
65547	0.03	0.03	0.03
68160	0.03	0.03	0.03
65317	0.03	0.03	0.03
64673	0.03	0.03	0.03
65933	0.03	1	0.03
65406	0.03	0.03	0.03
68246	0.03	0.03	0.03
65485	2	2	0.06
68164	2	2	0.06
68162	2	2	1
65411	0.03	0.03	0.03
65899	0.06	0.06	0.06
65321	2	2	2
63331	0.25	0.25	0.06
65408	2	2	0.125

**FIGURE 2:**
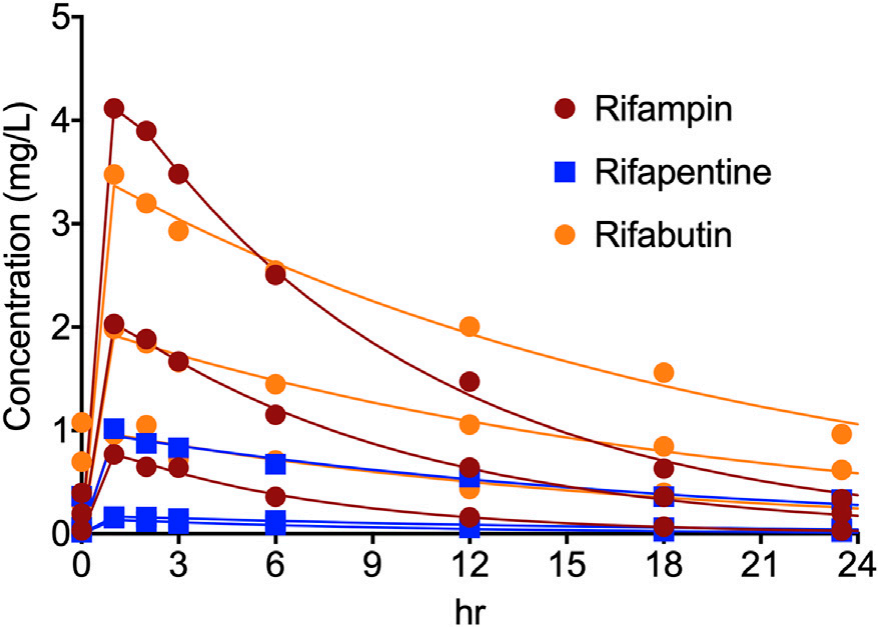
Concentration-time profile of rifampin, rifapentine, and rifabutin in the HFS-MAC. Pharmacokinetics model predicted concentration are shown as solid line and the symbol represent the observed concentrations in the HFS-MAS at each sampling time-point.

Figure 2 show the concentration-time profile of the rifampin, rifapentine, and rifabutin, as achieved in the HFS-MAC. The regression between model predicted vs. measured drug concentrations showed an *r* ^2^ of 0.997, indicating good model fit. The half-life of the drugs achieved in the HFS-MAC were 5.91 ± 0.98 h, 10.62 ± 3.03 h, and 13.29 ± 0.60 h for rifampin, rifapentine, and rifabutin, respectively. Since there was no protein present in the circulating media, Table 1 summarize the free (*f*)C_max_ and calculated drug exposure (*f*C_max_/MIC and *f*AUC_0–24_/MIC) of each drug in the HFS-MAC. To put the results in a clinical perspective, rifampin *f*C_max_ of 2.03 mg/L could be interpreted as a clinical dose of 600 mg per day, rifapentine *f*C_max_ of 1.02 mg/L equivalent to 1200 mg per day, and rifabutin *f*C_max_ of 1.05 mg/L equivalent to a 900 mg daily dose.

**FIGURE 3:**
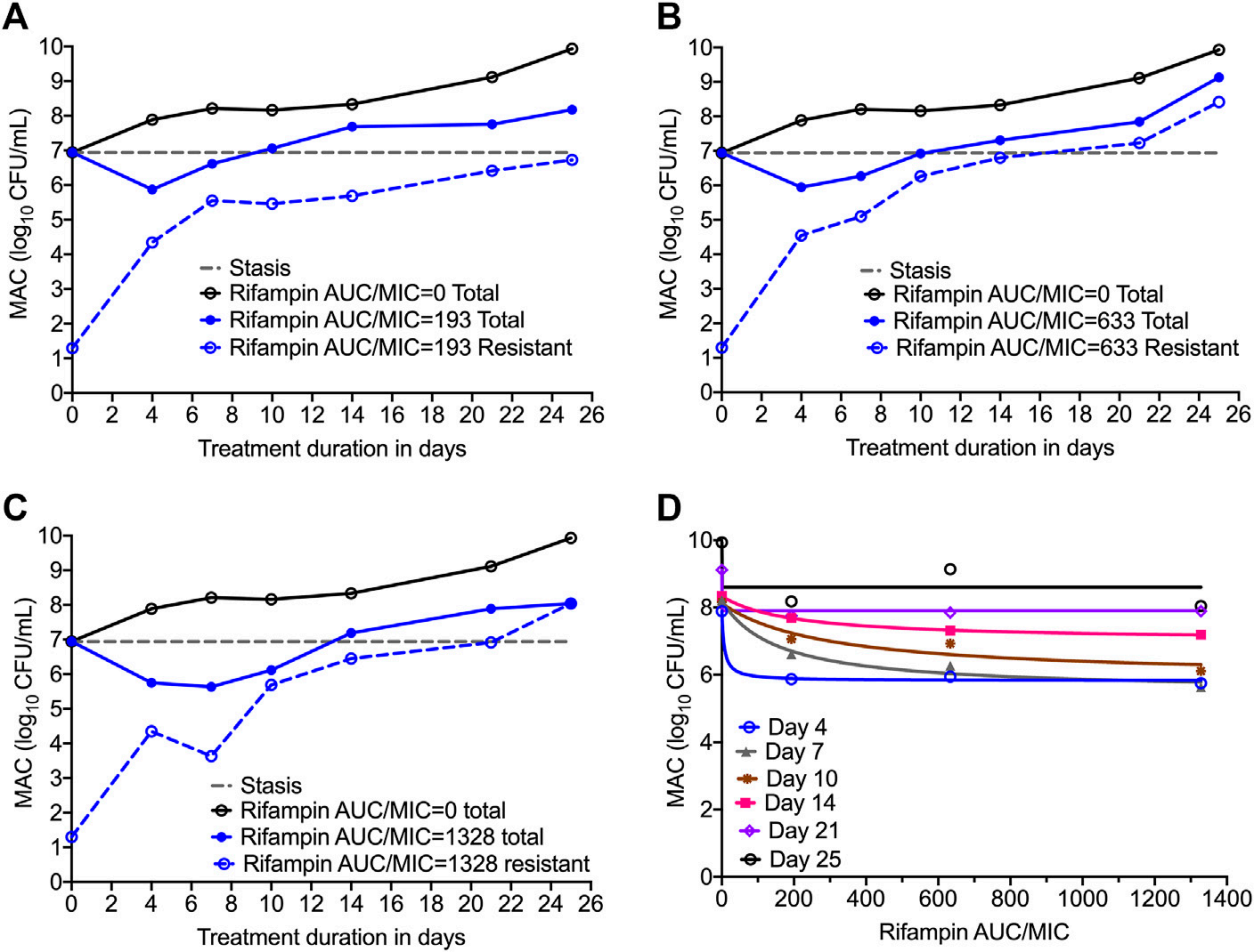
Efficacy of rifampin and resistance emergence in the HFS-MAC. **(A-C)**. All three rifampin exposures showed virtually identical kill of MAC for the first 4-days, then failed due to emergence of rifampin resistance in the HFS-MAC. The lowest rifampin exposure AUC/MIC = 193 while failed to control the emergence of drug resistance, had lowest drug-resistant subpopulation by the end of the study. **(D)**. Inhibitory Sigmoid E_max_ model to show the relationship between the rifampin exposure and bacterial burden. On day 14 [with highest *r* ^2^ = 0.999 and lowest Akaike Information Criteria score (Akaike, 1974) = 0.009], EC_50_ was calculated as AUC/MIC of 197.3.

Figures 3A–C show the time kill curves with different rifampin AUC/MIC exposures, and the emergence of resistance with time. At each rifampin monotherapy exposure there was a biphasic effect due to rapid emergence of resistance, with only a slight delay of 4 days at the highest rifampin AUC/MIC exposure. There was no statistically significant difference between the bacterial burden among the three rifampin exposures and ANOVA showed that the drug exposure could explain only 1.71% of the variance. Figures 3A–C show the change in the rifampin resistant subpopulation that was not statistically different from the total population (*p* > 0.05). Figure 3D and Table 3 show the inhibitory sigmoid E_max_ curves and parameters at different rifampin exposures for each sampling timepoint.

**TABLE 3:** Rifampin pharmacokinetics/pharmacodynamics indices for microbial kill in the HFS-MAC.

Study day	E_con_ log_10_ CFU/mL		E_max_ log_10_ CFU/mL		EC_50_ *f*AUC/MIC		*r* ^2^
	Estimate	Standard error	Estimate	Standard error	Estimate	Standard error	
4	7.89	0.13	2.07	0.19	6.65	20.64	0.99
7	8.20	0.29	2.69	0.50	154.66	110.15	0.98
10	8.12	0.41	2.23	0.88	294.18	390.79	0.92
14	8.33	0.02	1.32	0.03	197.34	16.58	1.00
21	9.11	0.10	1.26	0.14	0.00	74.35	0.99
26	9.92	0.84	1.46	1.16	0.00	173.21	0.70

**FIGURE 4:**
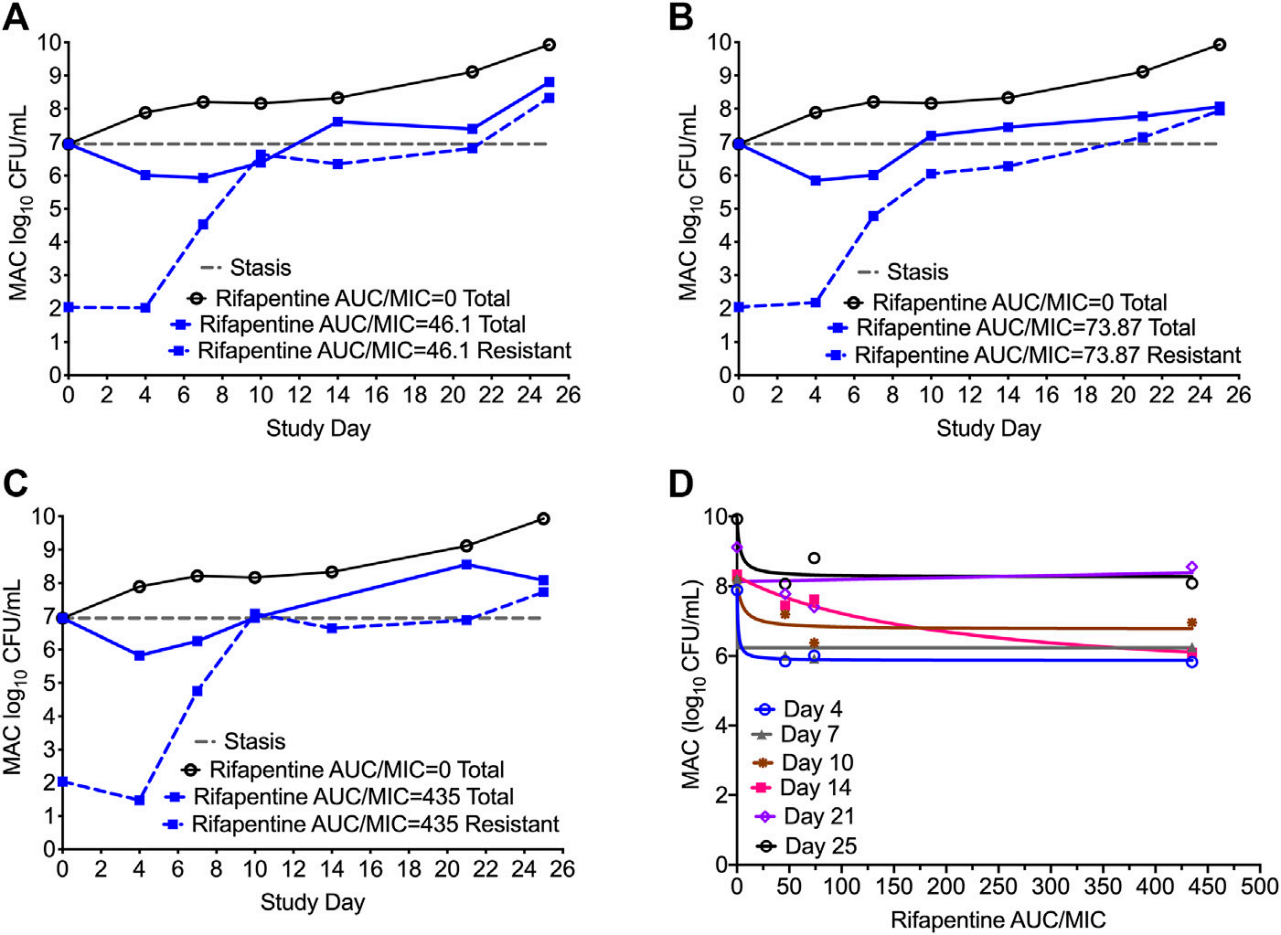
Rifapentine kill curve and resistance emergence in the HFS-MAC. **(A-C)**. There was no difference in the total as well as drug resistant subpopulation among the three rifapentine exposures tested in the HFS-MAC. The rifapentine monotherapy failed after 4 days of treatment. **(D)**. Inhibitory Sigmoid E_max_ model showed highest *r* ^2^ = 0.993 and lowest Akaike Information Criteria score (Akaike, 1974) = 0.080 on study day 4, where EC_50_ was calculated as AUC/MIC of 1.025.

Figures 4A–C show the time kill curve with different rifapentine AUC/MIC exposures, and emergence of resistance with time. Figure 4D and Table 4 show the inhibitory sigmoid E_max_ curves and parameters at different rifampin exposures. Figures 4A–C show the change in the MAC bacterial burden in response to the different rifapentine doses over time. The mean bacterial kill with the three rifapentine exposures during the first 4-days was 1.05 (95% CI: 0.81 to 1.29) log_10_ CFU/mL below stasis and the kill rate was −0.28 ± 0.03 log_10_ CFU/mL/day. However, as shown in Figures 4A–C, all three rifapentine exposure failed to control the growth of bacteria in the HFS-MAC, and the entire bacterial population became rifapentine resistant after just 26 days of the monotherapy. The difference in the total vs. drug resistant population was not statistically different [*p* > 0.05]. Figure 4D show the inhibitory sigmoid E_max_ curves with different rifapentine exposures for each sampling timepoint and Table 4 summarize the extent of bacterial kill and other model parameters for rifapentine performance in the HFS-MAC.

**TABLE 4:** Rifapentine pharmacokinetics/pharmacodynamics indices for microbial kill in the HFS-MAC.

Study day	E_con_ log_10_ CFU/mL		E_max_ log_10_ CFU/mL		EC_50_ *f*AUC/MIC		*r* ^2^
	Estimate	Standard error	Estimate	Standard error	Estimate	Standard error	
4	7.88	0.13	2.02	0.20	1.02	5.18	0.99
7	8.21	0.38	1.97	0.56	118.38	0	0.96
10	8.16	0.58	1.40	0.87	2318.36	4.95	0.79
14	8.28	0.30	3.14	1.00	50.39	0.00	0.96
21	8.13	0.66	0.00	0.00	50.32	0.00	0.02
26	9.93	0.59	1.67	0.88	0.00	2.89	0.85

**FIGURE 5:**
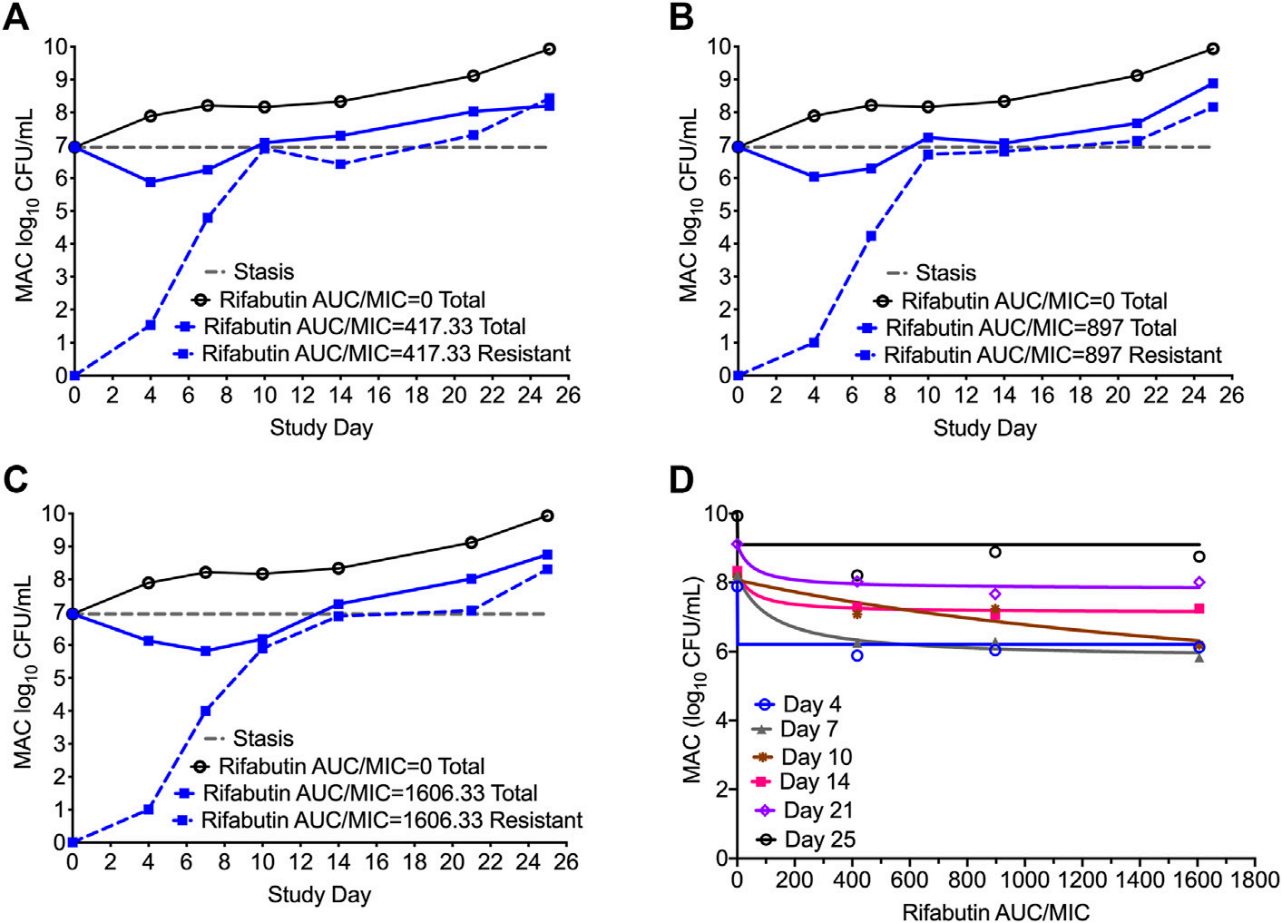
Rifabutin monotherapy in the HFS-MAC and resistance emergence **(A-C)**. Kill curve and emergence of rifabutin resistance with each of the three exposures tested in the HFS-MAC. Though there were no pre-existing rifabutin resistance colonies recorded in the inoculum, monotherapy led to rapid rifabutin resistance and entire MAC population was rifabutin resistant on day 25 **(D)**. Relationship between the rifabutin exposure and bacteria burden using the inhibitory sigmoid E_max_ model. The EC_50_ was calculated as AUC/MIC of 118.4 on day 7 of the study.

In Figures 5A–C we show the time kill curves with different rifabutin AUC/MIC exposures, as well as emergence of rifabutin resistance over 26 days study period. The overall kill, with the three rifabutin exposures, during the first 4 days of therapy was 0.9 (95% CI: 0.61 to 1.24) log_10_ CFU/mL below stasis with a kill rate of 0.23 ± 0.03 log_10_ CFU/mL/day. Despite there was no pre-existing rifabutin resistant subpopulation in the inoculum, we noticed rapid emergence of rifabutin resistance in the HFS-MAC. The relationship between the different rifabutin exposures and bacterial burden could be explained in Figure 5D and Table 5 summarize the other model parameters as calculated in the HFS-MAC. Table 6 summarize each rifamycin drug exposure, E_max_, kill slopes, and difference in the total and drug resistant MAC sub-population as achieved in the HFS-MAC.

**TABLE 5:** Rifabutin pharmacokinetics/pharmacodynamics indices for microbial kill in the HFS-MAC.

Study day	E_con_ log_10_ CFU/mL		E_max_ log_10_ CFU/mL		EC_50_ *f*AUC/MIC		*r* ^2^
	Estimate	Standard error	Estimate	Standard error	Estimate	Standard error	
4	7.88	0.37	1.68	0.58	0.00	171.80	0.94
7	8.20	0.26	2.41	0.47	118.38	141.94	0.97
10	8.06	0.50	4.29	8.25	2318.36	7350.63	0.86
14	8.33	0.15	1.21	0.26	50.39	125.52	0.97
21	9.11	0.27	1.30	0.46	50.32	209.32	0.93
26	9.93	0.98	0.83	1.53	0.00	904.01	0.38

**TABLE 6:** Comparison of kill slopes and bacterial burden in HFS-MAC treated with different rifamycins.

Drug exposure (*f*AUC/MIC)	Kill between day 0–4 (log_10_ CFU/mL)	Kill slope for day 0–4 (log_10_ CFU/ml/day)	Difference with non-treated control (log_10_ CFU/mL)	Difference between total and drug-resistant population (log_10_ CFU/mL)
Rifampin 192.97	1.07	−0.27	1.76	1.45
Rifampin 633.33	1.00	−0.25	0.80	0.71
Rifampin 1328	1.19	−0.30	1.89	0.00
Rifapentine 46.1	1.10	−0.27	1.87	0.12
Rifapentine 73.87	0.93	−0.23	1.13	0.47
Rifapentine 435	1.12	−0.28	1.85	0.35
Rifabutin 417.33	1.06	−0.27	1.73	−0.23^a^
Rifabutin 897	0.90	−0.23	1.05	0.72
Rifabutin 1606.33	0.81	−0.20	1.18	0.45

^a^ Negative value show that the drug resistant population replaced entire drug susceptible population.

## Discussion

While there are guidelines and recommendations in place, the treatment of pulmonary MAC is not straight forward and treatment failure as well as relapse is common. Moreover, in a recent study Griffith et al. (Griffith and Aksamit, 2020) argued that the “actual adherence to the guideline-based treatment approach by the clinicians is low”. In the recent years there has been attempts to optimize the treatment of MAC using the principles of PK/PD (Deshpande et al., 2010a; Deshpande et al., 2010b; Deshpande and Gumbo, 2011; Deshpande et al., 2016a; Deshpande et al., 2016b; Deshpande et al., 2017a; Deshpande et al., 2017b; Deshpande et al., 2017c; Deshpande et al., 2017d; Srivastava et al., 2017a; Srivastava et al., 2017b), as summarized in Table 7. However, the applicability of the PK/PD indices in the treatment of MAC remains to be understudied. Rifamycins are one of the recommended class of drug in the MAC combination regimen. However, pre-clinical PK/PD studies comparing rifampin, rifapentine, and rifabutin for MAC bacterial kill and resistance suppression are lacking. Such studies are important especially when new drug/drug delivery methods are being developed for MAC treatment and swapping of one rifamycin with other is recommended to prevent drug resistance (Griffith and Aksamit, 2020).

**TABLE 7:** Pharmacokinetics/pharmacodynamics parameters of different drug against MAC.

Drug	PK-PD index	E_max_	Kill below stasis	EC_80_	Stasis exposure	1.0 log_10_ CFU/mL exposure	2.0 log_10_ CFU/mL exposure
Azithromycin Deshpande et al. (2016a)	AUC_0-24_/MIC	2.11	0.6	3.43	1.29	NA	NA
Ethambutol Deshpande et al. (2010a)	C_max_/MIC	0.79	NA	1.23	NA	NA	NA
Moxifloxacin Deshpande et al. (2010b)	AUC_0-24_/MIC	3.03	3.0	163.37	6.0	17.12	391.56
Thioridazine Deshpande et al. (2016b)	C_max_/MIC	7.19	5.38	0.88	0.35	0.45	0.5
Linezolid Deshpande et al. (2017b)	AUC_0-24_/MIC	2.1	1.06	42	7.82	40.83	NA
Tidezolid Deshpande et al. (2017d)	AUC_0-24_/MIC	3.78	2.07	21.71	17.15	23.46	37.5
CAV Deshpande et al. (2017a)	%T_MIC_	2.71	2.4	52	9.0	70	100
Tigecycline^a^	AUC_0-24_/MIC	3.87	4.94	59	6.0	10.93	33.74

^a^ Unpublished data; CAV, ceftazidime-avibactam; E_max_ and kill below stasis represent log_10_ CFU/mL; stasis, initial bacterial burden; NA, Not achieved.

In the present study, we compared the three rifamycins mimicking human-like PK of each drug, and all three rifamycins failed to control the MAC growth despite showing initial bacterial kill, which was not significantly different from each other. The emergence of acquired drug resistance to the three rifamycins was also very similar and did not differ between the three drug exposures. We noticed that while the rifampin AUC/MIC = 192.97 exposure failed to kill MAC, had the lowest drug resistant subpopulation compare to the high drug exposures. Among the three rifamycins, there are clinical studies reporting rifampin is well tolerated in patients at dose up to 2400 mg/day (Zurr et al., 2016). Thus, formal dose-response studies are warranted to determine if higher rifampin exposure can overcome the resistance emergence. Further, the rifamycin’s MIC of the laboratory strain used in the studies was 0.032 mg/L, whereas the MIC_90_ for the clinical strains was substantially high. The probability of achieving optimal drug exposure decrease with increase in MIC. Thus, present results may be overoptimistic regarding rifamycin’s performance in the clinical setting.

Our study has limitations. First, we show that the rifamycins failed to control the bacterial growth when tested as monotherapy. The treatment of MAC pulmonary infection is a combination of drugs where drugs may protect each other in terms of resistance emergence. However, PK/PD studies with ethambutol and azithromycin show minimal efficacy of these drugs (Deshpande et al., 2010a; Deshpande et al., 2016a). Therefore, more effective drugs need to be explored/developed for successful treatment outcome. Second, we tested only three different doses of each rifamycin for efficacy and resistance suppression in MAC. Such study design may not accurately determine the optimal drug exposure for maximal kill and resistance suppression. Third, since rifapentine and rifabutin have long half-life compare to rifampin, dose-fractionations studies are required to determine the optimal dosing schedule to achieve the maximum efficacy, assuming the high dose will not be associated with severe adverse event, in combination of other effective anti-MAC drugs. Finally, the HFS-MAC lacks the complex immune system that may also play role in therapy outcome.

In summary, rifampin, rifapentine and rifabutin have comparable efficacy against MAC in the *in vitro* HFS-MAC model, and all three fail to control the MAC growth despite showing early bactericidal activity. The optimal rifamycin exposure for MAC kill remains to be determined.

## Data Availability

The original contributions presented in the study are included in the article/Supplementary material, further inquiries can be directed to the corresponding author.
